# Editorial: Machine learning in data analysis for stroke/endovascular therapy

**DOI:** 10.3389/fneur.2023.1267655

**Published:** 2023-08-24

**Authors:** Ari Ettleson, Benjamin Yim, Daniel A. Donoho

**Affiliations:** ^1^The George Washington University School of Medicine and Health Sciences, Washington, DC, United States; ^2^East Bay Brain and Spine Medical Group, Walnut Creek, CA, United States; ^3^Division of Neurosurgery, Center for Neuroscience, Children's National Medical Center, Washington, DC, United States

**Keywords:** stroke, neurointerventional surgery, machine learning, computer vision, endovascular treatment

## Introduction

Despite current methods of treatment and prevention, ischemic stroke causes more than 7 million deaths each year worldwide (1). Because of its prevalence, stroke research necessitates large data sets with numerous variables. Analysis of massive multivariable datasets has historically been unfeasible. Machine learning (ML) offers a paradigm-shifting opportunity to integrate several modes of data from larger cohorts, driving stroke research forward through its capacity to unravel complex relationships within intricate datasets.

In this Research Topic of *Frontiers in Neurology*, we called for original work on the theme of ML in endovascular therapy and stroke to collect novel approaches that may yield discovery.

Residual disability after stroke significantly diminishes stroke survivor quality of life, so novel and innovative technologies may be able to improve care. A pilot study by Weisinger et al. found that frequency-tuned electromagnetic field therapy can improve stroke motor function. These field therapies might be the basis of future products available in the clinic or new avenues of research to better understand the relationship between post-ischemia injury and functional connectivity.

The articles featured in this issue demonstrate the transformative potential of ML through a consistent approach: training ML models on a subset of retrospective clinical data and harnessing their predictive power. Li F. et al., for example, trained five algorithms to predict cerebral hemorrhage. The algorithm with the best performance revealed LDL, HDL, CRP, and Hgb as the strongest predictors. Wang et al.'s study trained six different ML models on a subset of data from patients hospitalized with acute ischemic stroke. Each algorithm identified significant predictors of death at 1-year post-stroke. The most successful algorithm was then used to build an ML network calculator—essentially a smart calculator—to identify high-risk patients. Models like these are life-saving alternatives to calculators like the NIH stroke scale or CHA2DS2–VASc scores.

Advanced clinical decision-making tools can be valuable in managing rare stroke subtypes, such as corpus callosum (CC) infarction. ML thrives on extracting patterns and insights from limited datasets, rendering its application even more significant given the inherent data scarcity in such cases. Xu et al.'s prospective analysis of CC infarction does exactly that. Even from a small cohort (*N* = 213) of CC infarctions, they were able to identify a logistic regression model that predicted subjective cognitive decline. Predicting cognitive decline may be useful in both prognostication and the development of specific rehabilitation paradigms. Li Q. et al.'s article, also featured in this issue, similarly uses ML as an assessment tool for post-stroke patients. Their meta-analysis included over 70,000 patients and demonstrated the ability of ML models to predict poor motor function after stroke.

Ye et al.'s article goes beyond these “smart calculators” by integrating clinical data with radiomic features. Their innovative approach improved prognosis prediction over models with only clinical or radiologic data. Approaches that integrate automated image analysis with clinical data have the potential to facilitate clinical decision-making about complex, high-risk stroke patients.

Beyond ML analysis of retrospective datasets to isolate predictors of stroke outcome, this issue also exemplifies the potential for image-based research, particularly advanced imaging modalities such as CT and MR. This approach, demonstrated by Kis et al.'s work, extends to studies by Werdiger et al. and Tetteh et al.'s, and can uncover insights into stroke therapy that cannot be seen without ML. Werdiger et al.'s study trained ML models on CT perfusion images to identify areas of ischemia. Clinically available software that identifies ischemic tissue relies on only one input, whereas here, multiple inputs led to higher accuracy. Tetteh et al.'s study—which also trained ML models on perfusion studies—used MR perfusion data, instead of CT. Their approach performed comparably to expert graders at identifying the quality of collateral circulation during stroke. Li Y. et al.'s work included a similar structure, except this time in the form of serial fMRI scans. Images were introduced to a support vector machine, somewhat of a supervised ML model, and researchers were able to compare outputs from stroke patients, healthy patients, and their respective follow-ups. Their analysis allowed them to “see” improvements in functional neural homotopy.

In addition to original research on stroke, this Research Topic also called for projects that leveraged ML techniques to investigate endovascular therapies. Risk reduction in endovascular surgery is particularly ripe for discovery by ML models. Endovascular thrombectomy (EVT), commonly performed to treat ischemic stroke, carries a significant risk of bleeding and thrombosis, making selection criteria paramount to risk mitigation. Kis et al., whose study is featured in this issue, combined automated analysis of pre-EVT CT images with clinical data to predict outcomes and improve prognostication after EVT. These findings demonstrate the need to integrate software-based analysis with clinical data when determining the futility of high-risk procedures like EVT.

As the landscape of stroke care continues to evolve, capturing intraoperative data becomes imperative to the modernization process. This primarily involves capturing the data in the form of surgical video and the application of computer vision techniques to analyze it. Computer vision, a type of task ML algorithms can be trained to complete, can analyze video-guided surgical procedures to reveal patterns and make nuanced predictions. Although endovascular therapy is not guided by high-resolution cameras, surgeons heavily rely on fluoroscopy. Other fields—laparoscopy and endoscopy—have already begun to capture, transmit, and analyze surgical video datasets using ML. Endovascular surgery lags behind.

Visual datasets—regardless of surgical field—are ripe for clinical discovery and generally under-exploited. Fluoroscopic runs are typically used for medical/diagnostic purposes. We (two practicing neurosurgeons, one of whom is a dual-trained cerebrovascular expert, and a medical student) have performed hundreds of angiograms; rarely do neurointerventionalists store angiographic data for systematic or quantitative analysis. Fluoroscopic images from endovascular procedures remain an untapped area of analysis. Existing research on barium swallow has demonstrated ML's ability to extract valuable information from videofluoroscopic datasets (2). ML analysis of fluoroscopic video from endovascular procedures is feasible, but to analyze the data we must first hit record.

In this editorial, we argue for the potential applications of computer vision in cerebrovascular care. In doing so, we described the current state of ML in stroke care, as well as its shortcomings. Using current examples of computer vision in surgical analysis, we will argue for the storage and analysis of fluoroscopic videos and images.

## Areas with potential

The first area with potential for application of ML is skill assessment. After fellowship, neurointerventional surgeons are expected to self-regulate their improvement from advanced to master. After fellowship, however, their overseers are no longer present to provide consistent, constructive feedback. Attending surgeons have little to base their performance on. Comparing complication or reoperation rates with national averages are the extent of their limited options for self-assessment.

ML models have begun to perform accurate surgical skill assessments in fields like urology and skull base surgery. A model trained on videos of radical prostatectomy was able to accurately assess a surgeon's ability to perform a specific step within the procedure (3). In this study, measurements were verified by corroborating them with expert grading of the same videos. The model was even able to mitigate bias created by small differences in video quality between institutions included in the study.

ML models have also correctly predicted a surgeon's ability to control blood loss during a lifelike surgical hemorrhage simulator (4). After being shown a brief video clip of the surgeons operating to stop bleeding, these models were better able to predict a surgeon's performance than expert reviewers. Imagine this same study structure, but replace the endoscopic video with recorded fluoroscopic runs of thrombectomies. Perhaps an ML system could view a proceduralist's ability to perform the beginning of a procedure and predict whether they would accomplish successful revascularization. Alternatively, an ML system might understand a local practitioner's prior skillset and be able to better predict whether they should first attempt a blood vessel-opening procedure in their local setting, perhaps with a tele-coach, or transfer the patient to a center of excellence.

Video data analysis can simplify complex surgical decision-making. Internal carotid artery stenosis, estimated to cause almost 10% of all ischemic strokes, can be treated with a multitude of procedures including endarterectomy, transfemoral carotid artery stenting (TF-CAS), or emerging, perhaps safer, technologies like transcarotid artery revascularization (TCAR) (5). At present, treatment allocation depends upon the judgment of the practitioner with few guidelines and highly variable utilization practices. AI models trained on clinical variables and fluoroscopic video might help surgeons to make informed decisions.

## Call to action: building video data repository for stroke and cerebrovascular care

The quantity of periprocedural cerebrovascular data left on the table is staggering. Consider the aforementioned removal of stroke-inducing plaques from the carotid artery, whether by surgical endarterectomy or percutaneous angioplasty and stenting, performed more than 100,000 times each year (6). Those procedures generate petabytes of angiographic and intraoperative visual data. Other fields that rely heavily on high-resolution cameras to guide procedures are already creating large, publicly available surgical video repositories for research and innovation. A large multimodal data repository that includes pre-, intra-, and post-procedural images as well as labeled clinical data could shed new light on the challenges of cerebrovascular and stroke care.

## Author contributions

AE: Conceptualization, Writing—original draft. BY: Supervision, Writing—review and editing. DD: Supervision, Writing—review and editing.

## References

[1] GBD 2019 Stroke Collaborators. Global, regional, and national burden of stroke and its risk factors, 1990–2019: a systematic analysis for the global burden of disease study 2019. Lancet Neurol. (2021) 20:795–820. 10.1016/S1474-4422(21)00252-0 34487721 PMC8443449

[2] Kim JK Choo YJ Choi GS Shin H Chang MC Park D. Deep learning analysis to automatically detect the presence of penetration or aspiration in videofluoroscopic swallowing study. J Korean Med Sci. (2022) 37:e42. 10.3346/jkms.2022.37.e42 35166079 PMC8845107

[3] Kiyasseh D Laca J Haque TF Miles BJ Wagner C Donoho DA. A multi-institutional study using artificial intelligence to provide reliable and fair feedback to surgeons. Commun Med. (2023) 3:42. 10.1038/s43856-023-00263-3 36997578 PMC10063640

[4] Pangal DJ Kugener G Zhu Y Sinha A Unadkat V Cote DJ. Expert surgeons and deep learning models can predict the outcome of surgical hemorrhage from 1 min of video. Sci Rep. (2022) 12:8137. 10.1038/s41598-022-11549-2 35581213 PMC9114003

[5] Flaherty ML Kissela B Khoury JC Alwell K Moomaw CJ Woo D. Carotid artery stenosis as a cause of stroke. Neuroepidemiology. (2013) 40:36–41. 10.1159/000341410 23075828 PMC3626492

[6] Maini BS Mullins TF III Catlin J O'Mara P. Carotid endarterectomy: a ten-year analysis of outcome and cost of treatment. J Vasc Surg. (1990) 12:732–40. 10.1067/mva.1990.25015 2243409

